# Mapping the epigenetic modifications of DNA and RNA

**DOI:** 10.1007/s13238-020-00733-7

**Published:** 2020-05-22

**Authors:** Lin-Yong Zhao, Jinghui Song, Yibin Liu, Chun-Xiao Song, Chengqi Yi

**Affiliations:** 1 Department of Gastrointestinal Surgery and Laboratory of Gastric Cancer, State Key Laboratory of Biotherapy, West China Hospital, Sichuan University, and Collaborative Innovation Center for Biotherapy, Chengdu, 610041 China; 2 Ludwig Institute for Cancer Research, Nuffield Department of Medicine, University of Oxford, Oxford, OX3 7FZ UK; 3 Target Discovery Institute, Nuffield Department of Medicine, University of Oxford, Oxford, OX3 7FZ UK; 4 State Key Laboratory of Protein and Plant Gene Research, School of Life Sciences, Peking University, Beijing, 100871 China; 5 Peking-Tsinghua Center for Life Sciences, Peking University, Beijing, 100871 China; 6 Department of Chemical Biology and Synthetic and Functional Biomolecules Center, College of Chemistry and Molecular Engineering, Peking University, Beijing, 100871 China

**Keywords:** DNA modification, DNA methylation, RNA modification, epitranscriptomics, epigenetics, long read sequencing

## Abstract

Over 17 and 160 types of chemical modifications have been identified in DNA and RNA, respectively. The interest in understanding the various biological functions of DNA and RNA modifications has lead to the cutting-edged fields of epigenomics and epitranscriptomics. Developing chemical and biological tools to detect specific modifications in the genome or transcriptome has greatly facilitated their study. Here, we review the recent technological advances in this rapidly evolving field. We focus on high-throughput detection methods and biological findings for these modifications, and discuss questions to be addressed as well. We also summarize third-generation sequencing methods, which enable long-read and single-molecule sequencing of DNA and RNA modification.

## Introduction

Mapping the epigenetic modifications of DNA and RNA becomes increasingly crucial to understand their diverse biological functions. At least 17 and 160 types of chemical modifications have been discovered in DNA and RNA, respectively (Raiber et al., 2017; Boccaletto et al., 2018). DNA modification plays important roles in several biological processes and diseases, including development (Greenberg and Bourc’his, 2019), aging (Unnikrishnan et al., 2019), cancer (Koch et al., 2018), etc. These modifications would not interfere with Watson-Crick pairing but affect the DNA-protein interaction while in the major groove of the double helix. In the mammalian genome, methylation at the 5th carbon of cytosine (5-methylcytosine, or 5mC) is the most predominant DNA modification, which is also called the “fifth base” (Greenberg and Bourc’his, 2019). The reaction is catalyzed by DNA methyltransferases (DNMTs) and mostly found in the context of symmetrical CpG dinucleotides, although a small percentage of methylation at CHG and CHH sequences (where H correspond to A, T or C) is also observed in embryonic stem (ES) cells. While showing tissue-specific differences, mammalian genomes exhibit particularly high CpG methylation levels, 70% to 80% of CpGs are methylated (Li and Zhang, 2014).

Other modifications apart from 5mC have also been found in mammalian DNA. In 2009, two groups have independently reported the existence of 5-hydroxymethylcytosine (5hmC) in mammalian genome, which is now widely accepted as the “sixth base”. Tahiliani et al. showed that the ten-eleven translocation 1 (TET1) enzyme catalyses the conversion of 5mC to 5hmC (Tahiliani et al., 2009), while Kriaucionis demonstrated the presence of 5hmC in mouse brain (Kriaucionis and Heintz, 2009). Further successive oxidations mediated by TET result in formation of 5-formylcytosine (5fC) and 5-carboxylcytosine (5caC) (He et al., 2011; Ito et al., 2011; Pfaffeneder et al., 2011). These two oxidative products are hypothesized to be intermediates in an active DNA demethylation pathway, which are excised by thymine DNA glycosylase (TDG) and restored to unmodified cytosines through the base excision repair (BER) pathway (He et al., 2011).

Similar to DNA, cellular RNA is also decorated with diverse chemical modifications, and such modifications participate in all aspects of RNA metabolism. The multitude of modifications in RNA add a new layer to the gene regulation, leading to the emerging field of “RNA epigenetics” or “epitranscriptomics” (He, 2010; Frye et al., 2016; Gilbert et al., 2016; Roundtree et al., 2017). Recently developed high-throughput sequencing technologies for detecting RNA modifications have greatly accelerated the functional study of epitranscriptomics (Li et al., 2016b). Here, we primarily focus on mRNA modifications, including N^6^-methyladenosine (m^6^A), N^6^,2’-O-dimethyladenosine (m^6^Am), N^1^-methyladenosine (m^1^A), 5-methylcytosine (m^5^C), 5-hydroxymethylcytosine (hm^5^C), N^4^-acetylcytidine (ac^4^C), pseudouridine (Ψ), N^7^-methylguanosine (m^7^G), etc.

**Figure 1:**
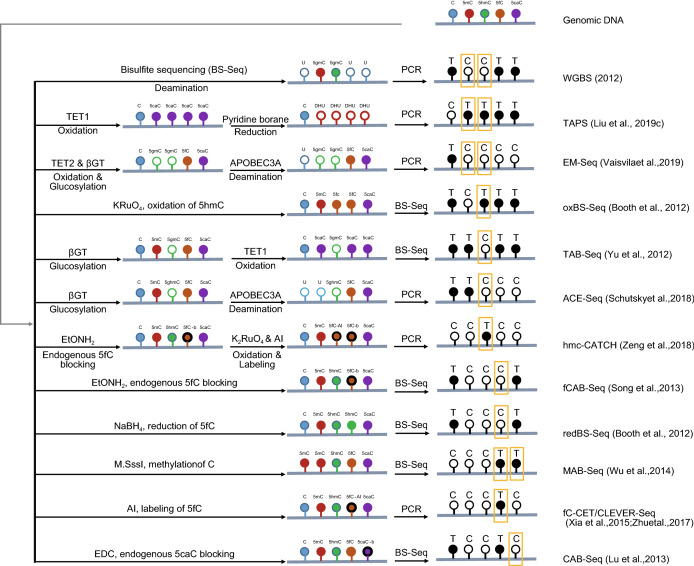
**Single-base resolution methods for quantitatively profiling mammalian DNA modifications of cytosine**. For 5 methylcytosine (5mC) mapping there are three methods, whole-genome bisulfite sequencing (WGBS), TET-assisted pyridine borane sequencing (TAPS) and enzymatic methyl-sequencing (EM-Seq); For 5-hydroxymethylcytosine (5hmC) mapping in single-base resolution there are four methods, oxidative bisulphite sequencing (oxBS-Seq), TET-assisted bisulphite sequencing (TAB-Seq) and APOBEC-coupled epigenetic sequencing (ACE-Seq) as well as chemical-assistant C-to-T conversion of 5hmC sequencing (hmC-CATCH); Four sequencing methods for mapping the 5-formylcytosine (5fC), chemically assisted bisulfite sequencing (fCAB-Seq), reduced BS-Seq (redBS-Seq), *M.SssI* methylase-assisted bisulfite sequencing (MAB-Seq) and 5fC cyclization-enabled C-to-T transition of 5fC (fC-CET); Chemical modification-assisted bisulfite sequencing (CAB-Seq) is a singe-base resolution sequencing method to map 5caC

**Figure 2:**
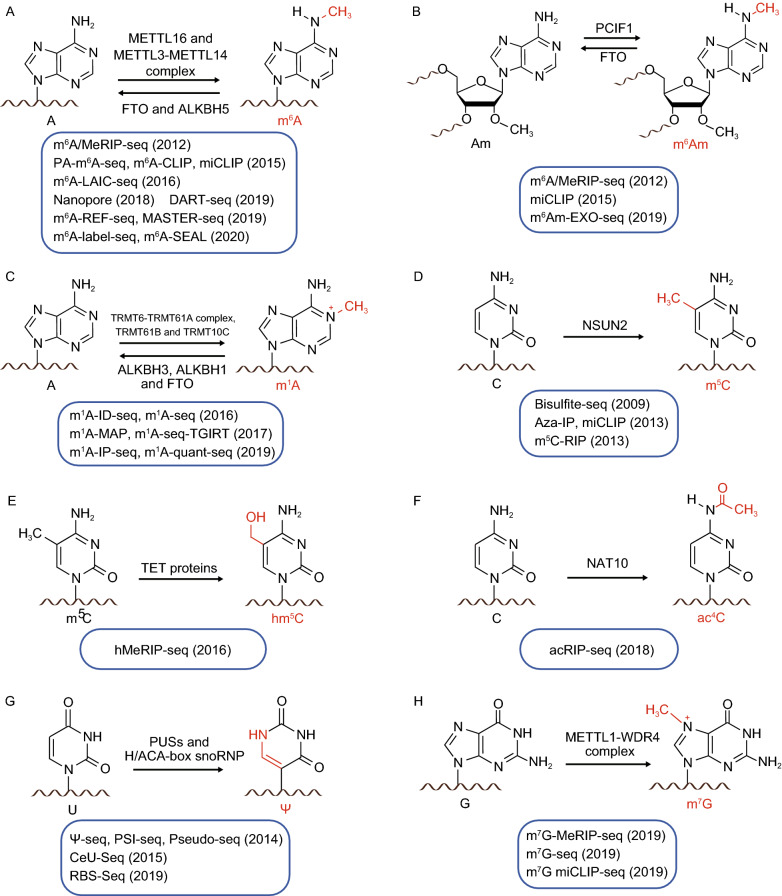
**Chemical structures, modification enzymes and high-throughput detection strategies of modifications in the transcriptome**

The interest in understanding the functions of DNA and RNA modifications as well as the related molecular mechanisms has been growing, which drives the progresses in developing chemical and biochemical tools to detect specific modifications within genomes and transcriptomes. On the other hand, the development of new technologies contributes to increased knowledge on modifications of DNA and RNA. In this review, we mainly focus on high-throughput detection strategies for DNA (Fig. 1) and RNA (Fig. 2) modifications, and their biological findings as well as questions to be addressed.

## Base-resolution sequencing for DNA modifications

### Base-resolution sequencing of the predominant DNA modification: 5mC

Bisulfite sequencing (BS-Seq), is regarded as the gold standard for 5mC detection. It is based on the differential reactivity of bisulfite between methylated C (5mC and 5hmC) and unmethylated C, in which DNA is treated with bisulfite that leads to the deamination of unmethylated C to Uracil (U) while methylated C is resistant to deamination. In the subsequent PCR amplification, methylated C remains C while unmethylated C can be readout as T. Whole-genome bisulfite sequencing (WGBS), as a whole genome method of BS-Seq, has been widely utilized in DNA methylation profiling, as it can provide single-base resolution with full genome coverage (Fig. 1). The readouts of methylated or unmethylated C from individual genomic locations of the whole genome are digital counts in WGBS, resulting in high resolution and precision with unmethylated C conversion efficiency over 99%, thus making WGBS the most accepted method for charting the DNA methylation landscape (Adey and Shendure, 2012; Kobayashi and Kono, 2012; Yamaguchi et al., 2012; Kobayashi et al., 2013; Shirane et al., 2013). However, the harsh bisulfite treatment degrades the majority of the DNA (Tanaka and Okamoto, 2007), which severely limits its applications to those precious DNA samples with low input, even though several efforts have been made to improve the DNA recovery (Smallwood et al., 2014; Clark et al., 2017). Moreover, since unmethylated cytosines accounting for nearly 95% of the total cytosine in mammalian genome are converted to thymine, the bisulfite treatment reduces the sequence complexity of template DNA, leading to low mapping rates, uneven genome coverages and inherent biases. The last but not the least it should be noted that bisulfite conversion could not distinguish between 5mC and 5hmC, given that it only provides the combined signal of 5mC and 5hmC.

Recently, two bisulfite-free whole-genome base-resolution DNA methylation sequencing methods have been developed to replace WGBS. The TET-assisted pyridine borane sequencing (TAPS) (Liu et al., 2019b) was introduced to detect 5mC and 5hmC, in which 5mC and 5hmC are firstly oxidized by TET to 5caC and then reduced to dihydrouracil (DHU) using pyridine borane with around 98% conversion efficiency, and subsequently readout as T after PCR amplifications (Fig. 1). It should be noted that the conversion rates of TAPS and bisulfite sequencing are different measurements. When comparing like-for-like, TAPS has a lower false positive rate (falsely detect unmodified C as modified, 0.23%) than bisulfite sequencing (0.6%). Compared with bisulfite sequencing, TAPS further demonstrates higher mapping rate and quality, more even coverage as well as lower sequencing cost. Through mild enzymatic and chemical reactions, TAPS can work effectively with as little as 1 ng of genomic DNA and circulating cell-free DNA, illustrating its potential for the clinical applications on challenging samples. As a nondestructive sequencing method, TAPS can preserve DNA fragments over 10 kilobases long, based on which a targeted long-read TAPS (lrTAPs) was recently develped (Liu et al., 2020), allowing accurate long-range methylation sequencing and phasing with third-generation sequencing technologies, such as Nanopore and SMRT sequencing. Modification of TAPS such as addition of β-glucosyltransferase (βGT) protection or replacement of TET enzyme with potassium perruthenate for oxidation could enable selective sequencing of 5mC (TAPSβ) or 5hmC (chemical-assisted pyridine borane sequencing, CAPS) (Liu et al., 2019b).

Another bisulfite-free method, the enzyme-based method Enzymatic Methyl-Seq (EM-Seq) has been developed by New England BioLabs, which first utilizes TET2 and βGT to oxidize and glucosylate 5mC and 5hmC to 5gmC. This provides protection from deamination by the AID/APOBEC family DNA deaminase APOBEC3A in the next step while unmodified C is deaminated to U (Fig. 1). EM-Seq showed higher mapping efficiency and more uniform GC coverage than BS-Seq. However, conversion of all unmodified C to U by EM-Seq would still cause low complexicity problem in the sequencing library and lower DNA input such as 100 pg resulted in PCR duplicate rate as high as 84.5% while only 10.8% of the reads were usable (Vaisvila et al., 2019).

### Base-resolution sequencing of the “sixth base”: 5hmC

The in-depth investigation of the biological functions of 5hmC requires elucidating the distribution patterns of 5hmC in genomes, preferentially at single-nucleotide resolution. Two modified BS-seq methods has been developed for mapping 5hmC. Oxidative bisulfite sequencing (oxBS-Seq) (Booth et al., 2012) is based on the selective and quantitative chemical oxidation of 5hmC using potassium perruthenate (KRuO_4_) to produce 5fC that subsequently converted to U by bisulfite treatment with an overall 5hmC-to-U conversion rate of 94.5% (Fig. 1). The absolute level and precise position of 5hmC in oxBS-Seq will be detected by subtracting signals of oxBS-Seq from BS-Seq (mESCs) (Booth et al., 2012, 2013). Deep sequencing depth is required to achieve high-confidence 5hmC mapping for oxBS-Seq, as it needs subtraction from two random sampling-based BS-Seq experiments are required.

TET-assisted bisulfite sequencing (TAB-Seq) is an approach in which 5hmC is firstly modified using βGT, and then 5mC is subsequently oxidized to 5caC by TET1 (Fig. 1). Subsequent bisulfite treatment enables 5hmC detected as C, while C, 5mC, 5fC and 5caC are readout as T, offering a strategy for the directly mapping of 5hmC at a single base resolution (Yu et al., 2012a, b). TAB-Seq can achive over 96% of conversion rate of 5mC to T in genomic DNA with over 90% of 5hmC protected from conversion. It has been applied to not only confirm a high-confidence mapping of widespread distribution of 5hmC across the whole genome of mouse embryonic stem cells (mESCs) but also demonstrate strand asymmetry and sequence bias at the 5hmC. Additionally, 5hmC was shown to be highly enriched at distal regulatory elements through TAB-seq analysis.

More recently, two bisulfite-free approaches have been developed to map 5hmC at base-resolution. Chemical-assistant C-to-T conversion of 5hmC sequencing (hmC-CATCH) is a bisulfite-free method to map 5hmC, which is based on selective oxidation of 5hmC to 5fC by potassium ruthenate (K_2_RuO_4_) with a conversion efficiency of ~94% and subsequent chemical labeling and conversion of 5fC to T during PCR (Zeng et al., 2018) (Fig. 1). hmC-CATCH allows direct detection of 5hmC as T without affecting unmodified C or 5mC. It was illustrated that potassium ruthenate causes less DNA damage than potassium perruthenate, and enables the mapping of 5hmC with nanoscale genomic DNA, which is especially benificial for those biological and clinical samples with limited amounts. Futhermore, this method was applied to detect the cell-free DNA (cfDNA) of healthy donors and cancer patients, and revealed base-resolution hydroxymethylome in the human cfDNA for the first time.

Another method, APOBEC-coupled epigenetic sequencing (ACE-Seq) (Schutsky et al., 2018) has been developed as a bisulfite-free and enzymatic method for base resolution of sequencing of 5hmC (Fig. 1). Similar to EM-Seq, it uses AID/APOBEC to deaminate unmodified C and 5mC to U after protecting 5hmC with βGT first, so it remains as C after PCR amplification. ACE-Seq achieved 99.9% and 99.5% conversion rates for cytosine and 5mC, respectively, while 98.5% of 5hmC remained as C. Compared with conventional bisulfite-based methods, ACE-seq is non-destructive, which allows for high confidence 5hmC profiles with up to 1000-fold less DNA input. 5hmC was found to be almost entirely confined to CG dinucleotides in tissue-derived cortical excitatory neurons by using ACE-seq. Similarly, Li et al. reported an APOBEC3A-mediated deamination sequencing (AMD-seq) which was also established for localization analysis of 5hmC at base-resolution (Li et al., 2018).

### Base-resolution sequencing of 5fC

5fC chemically assisted bisulfite sequencing (fCAB-Seq) was the first quantitative method to sequence 5fC at single-base resolution in genomic DNA (Song et al., 2013) (Fig. 1). In fCAB-Seq, 5fC is modified with *O*-ethylhydroxylamine (EtONH_2_) to form a derivative which can not be converted to U during the following BS-Seq. Therefore, the precise genomic locations of 5fC at single-base level can be identified, through comparison of EtONH_2_-treated BS-Seq and conventional BS-Seq of the same sample. Applying fCAB-Seq, low abundance 5fC at endogenous loci at levels down to only a few percent could be detected. Another bisulfite-based method termed reduced BS-Seq (redBS-Seq) was developed to quantititively detect 5fC in genomic DNA at single-base resolution (Booth et al., 2014), which is based on a selective reduction of 5fC to 5hmC by sodium borohydride (NaBH_4_) followed by BS-Seq (Fig. 1). Using redBS-Seq, 5fC was demonstrated to be negatively correlated to 5hmC in locations where 5fC and 5hmC appeared simultaneously. The 5fC protection rate for fCAB-Seq is 50%–60%, while it is nearly 97% for redBS-Seq.

Another bisulfite-dependent genome-wide method, termed methylase-assisted bisulfite sequencing (MAB-Seq), can quantitatively detect 5fC and 5caC simultaneously at single-base resolution (Guo et al., 2014; Wu et al., 2014; Neri et al., 2015) (Fig. 1). In this approach, genomic DNA is first treated with the CpG methyltransferase M.SssI which efficiently methylates CpG dinucleotides, and the following bisulfite treatment can only result in deamination of 5fC and 5caC which readout as T, while C, 5mC and 5hmC are readout as C in the subsequently sequencing, since unmodified CpGs in the original genomic DNA are mythylated as 5mCpG. MAB-Seq, through which 84.7% of 5fC and 99.5% of 5caC are efficently converted, respectively, reveals strong strand asymmetry of active demethylation within palindromic CpGs. Using this method, 5fC and 5caC in ESCs were found to occur on active promoters and enhancers, and be associated with TET and TDG. The generation and excision of 5fC and 5caC indicated a dynamic DNA demethylation activity mediated by TET/TDG using MAB-Seq combined with *Tdg* depletion. MAB-seq could be further combined with sodium borohydride reduction to map 5caC and 5fC separately at a single base-resolution (Wu et al., 2016).

Two bisulfite-free sequencing methods have been developed to map 5fC at a single base-resolution in genomic scale. In fC-CET (5fC cyclization-enabled C-to-T transition), an azido derivative of 1,3-indandione (AI) was used to achieve selectively labelling of 5fC (Xia et al., 2015) (Fig. 1). The azide group in the labelling adduct enabled the efficient enrichment of 5fC containing DNA fragments, which largely reduced the sequencing cost for 5fC detection in a whole genome as compared with fCAB-Seq and redBS-Seq, considering the limited abundance of 5fC in the genome. With this method, genome-wide 5fC maps were obtained on the single-base level for the first time in both Tdg^fl/fl^ mESCs and Tdg^−/−^ mESCs with no noticeable DNA degradation, demonstrating a limited overlap with 5hmC. Moreover, the first single-cell 5fC sequencing method termed chemical-labeling-enabled C-to-T conversion sequencing (CLEVER-Seq) was introduced based on malononitrile labeling of 5fC (Zhu et al., 2017). With this method, conversion rate of ∼86.4% was observed for the 5fC site. Besides, the highly dynamic 5fC profile and its intrinsic heterogeneity were revealed at single base resolution for mouse embryos and mESCs, and the abundance of 5fC in promoter region could regulate corresponding gene expression.

### Base-resolution sequencing of 5caC

Chemical modification-assisted bisulfite sequencing (CAB-Seq) has been developed to sequence 5caC at base-resolation (Lu et al., 2013) (Fig. 1). In CAB-Seq, 5caC is protected as an amide in a 1-ethyl-3-[3-imethylaminopropyl] carbodiimide hydrochloride (EDC) catalyzed reaction, which could not be converted to U during bisulfite treatment, and hereby readout as C. Therefore, 5caC could be detected by subtracting the BS-Seq signal from CAB-seq method. Based on CAB-Seq, DNA immunoprecipitation-coupled CAB-Seq (DIP-CAB-Seq) (Lu et al., 2015), as a pre-enrichment-based bisulfite sequencing strategy, was developed to map 5fC and 5caC at single-base resolution level in genome-wide both for WT and Tdg KO mouse ESCs, and illustrated only a very limited overlap existed between 5fC and 5caC.

### Antibody- or immunoprecipitation (IP)- based mapping methods for modified DNA

While we focus on base-resolution sequencing methods, antibody- or IP-based DNA modification detection strategies are traditionally widely used for the sake of simple and low-cost features. Methylated DNA immunoprecipitation (MeDIP)(Weber et al., 2005) used a 5mC-specific antibody to recognize and pull-down the DNA fragment with 5mC modification. Similar to MeDIP, 5hmC/5fC/5caC, can be recognized with specific antibodies (Ficz et al., 2011; Stroud et al., 2011; Shen et al., 2013).

A method profiling 5hmC in genomic DNA termed as hmC-seal (Song et al., 2011b) was developed as an antibody-independent method on the basis of selective chemical labeling and the extremely specific and tight biotin-streptavidin interaction, which can be then used to perform selective pull-down. Using hmC-seal to profile 5hmC, researchers found 5hmC signatures in cell free DNA could be diagnostic biomarkers for human cancers (Li et al., 2017a; Song et al., 2017).

Despite the low-cost sequencing, the antibody- or IP- based methods for modified DNA are not quantitative and do not offer base-resolution information. In addition, the specificity is highly depended on the quality of the antibody, and high background noise could result from cross-reactivity with off-target sites and intrinsic affinity of IgG for short unmodified DNA repeats (Booth et al., 2015; Lentini et al., 2018). Therefore, profile of modified DNA detected by antibody-or IP-based methods should be interpreted with care.

### Sequencing of N^6^-methyladenine (6mA) in DNA

In spite of its scarcity in mammalian DNA, 6mA has grabed increasing attention since the presence of 6mA in various eukaryotic genomes was confirmed in 2015 (Fu et al., 2015; Greer et al., 2015; Zhang et al., 2015). LC-MS/MS mass spectrometry can quantify the proportion of 6mA/A with a high sensitivity and is able to detect 6mA with very low abundance. DNA 6mA sequencing mainly relies on antibody enrichment, which is prone to background noise and off-target binding as desbribed above (Lentini et al., 2018). The third generation sequencing methods are also used to identify 6mA in DNA, which are discussed in the third part. However, recent studies revealed that the sample contamination, RNA contamination, technological limitations, and antibody non-specificity may cause serious problems in quantification and sequencing of 6mA in mammalian genomic DNA, casting doubts on the significance of 6mA in the mammalian genome (O’Brown et al., 2019; Douvlataniotis et al., 2020; Musheev et al., 2020). However, 6mA could be a regulatory mark in mammalian mitochondrial DNA (mtDNA) (Hao et al., 2020).

## The biogenesis and sequencing approaches for RNA modifications

### The most prevalent internal mRNA modification: m^6^A

m^6^A is the most prevalent internal modification in eukaryotic mRNA. It is primarily catalyzed by a methyltransferase complex (termed “writers”) consisting of METTL3 and METTL14 as well as additional protein subunits (including WTAP, VIRMA, HAKAI, Zc3h13, and RBM15/15B) (Harper et al., 1990; Bokar et al., 1994; Liu et al., 2014; Ping et al., 2014; Schwartz et al., 2014b; Wang et al., 2014; Patil et al., 2016; Wen et al., 2018; Yue et al., 2018) (Fig. 2A). Another methyltransferase METTL16 has been identified to methylate *MAT2A* mRNA (Pendleton et al., 2017) (Fig. 2A). m^6^A can be demethylated by FTO and ALKBH5 (“erasers”) (Jia et al., 2011; Zheng et al., 2013) (Fig. 2A), hence is a reversible modification. Dynamic m^6^A methylomes have been identified in physiological processes, across tissues, and in response to stimuli (Dominissini et al., 2012; Meyer et al., 2012; Schwartz et al., 2013; Zhou et al., 2015; Cao and Li, 2016; Roundtree et al., 2017; Liu et al., 2019c; Xiao et al., 2019). As an important epitranscriptomic mark, m^6^A plays critical roles in mRNA splicing, polyadenylation, export, translation, stability, structure, etc.

Most of the high-throughput sequencing methods of m^6^A rely on an m^6^A-specific antibody. For instance, m^6^A/MeRIP-Seq uses the antibody to identify thousands of m^6^A peaks in mammalian mRNA (Dominissini et al., 2012; Meyer et al., 2012). PA-m^6^A-Seq, m^6^A-CLIP, and miCLIP utilize UV-induced antibody-RNA crosslinking to obtain the base-resolution m^6^A profiles (Chen et al., 2015; Ke et al., 2015; Linder et al., 2015). m^6^A-LAIC-Seq compares RNA abundances in m^6^A-positive and m^6^A-negative fractions to quantify the m^6^A stoichiometry on a transcriptome-wide scale (Molinie et al., 2016). Endoribonuclease-based strategies to detect m^6^A (MAZTER-Seq and m^6^A-REF-Seq) have been developed, providing examples of antibody-independent m^6^A sequencing methods (Garcia-Campos et al., 2019; Zhang et al., 2019b). Another antibody-free m^6^A sequencing method, DART-Seq, utilizes fused APOBEC1-YTH protein to induce C-to-U editing at site adjacent to m^6^A, thus identifying m^6^A sites (Meyer, 2019). Very recently, two chemical labeling methods (m6A-label-seq and m6A-SEAL) have also been developed (Shu et al., 2020; Wang et al., 2020). Despite the fact that m^6^A has been profiled extensively, cautions should still be taken when using specific methods for m^6^A detection. For instance, the antibody-based methods could be influenced by the intrinsic bias of the antibody and binding to particular RNA sequence or other modification (Schwartz et al., 2013; Linder et al., 2015). For the endoribonuclease-based methods, they do not pre-enrich m^6^A sites, have motif preference and thus detect only part of m^6^A sites. For the chemical labeling methods, labeling efficiency are needed to be improved. Hence, new methods are still desired to facilitate the study of m^6^A.

Despite these advances supporting the crucial roles of m^6^A in various cellular and physiological processes, there are still many issues in our understanding of m^6^A-mediated regulatory roles in gene expression. FTO, the first RNA demethylase identified both *in vivo* and *in vitro* to erase m^6^A, binds to exon and intron regions of pre-mRNA (Jia et al., 2011; Fu et al., 2013; Bartosovic et al., 2017). FTO-mediated demethylation of m^6^A has regulatory roles in alternative splicing and translation (Bartosovic et al., 2017; Yu et al., 2018). FTO dynamically regulates m^6^A RNA in response to heat shock stress, DNA UV damage and virus infection (Zhou et al., 2015; Gokhale et al., 2016; Xiang et al., 2017). Moreover, FTO-mediated m^6^A demethylation affects cell growth and plays an oncogenic role in cancer cells (Cui et al., 2017a; Li et al., 2017c; Su et al., 2018). Therefore, the demethylase activity of FTO is very important for diverse physiological processes. A recent study reported in a liver-specific Fto-transgenic mice model, Fto can mediate demethylation of both internal m^6^A and cap m^6^Am (Zhou et al., 2018). Moreover, another study found FTO preferentially demethylates m^6^Am than m^6^A (Mauer et al., 2017). Further investigations found that FTO shows differential substrate preferences for m^6^A and m^6^Am in polyadenylated RNA in the nucleus versus in the cytoplasm, and can mediate tRNA m^1^A demethylation as well (Wei et al., 2018). Collectively, FTO can demethylate multiple substrates, but it is still unclear how FTO coordinates the demethylation of multiple modifications and what are the regulatory roles of FTO in each methylation substrates.

m^6^A can play an important role in pre-mRNA splicing. An initial study has revealed that m^6^A peaks are overrepresented in alternative exons, suggesting m^6^A may have regulatory functions in mRNA splicing (Dominissini et al., 2012). Further investigations reported that perturbation of m^6^A writers, erasers, or readers has effects on splicing. For m^6^A writers, the depletion of Mettl3 in mouse embryonic stem cells (mESCs) significantly affects alternative splicing (Geula et al., 2015); METTL16 can modify *MAT2A* transcript and regulate intron retention of *MAT2A* (Pendleton et al., 2017). For m^6^A erasers, the depletion of ALKBH5 was shown to alter splicing in HeLa cells (Zheng et al., 2013); FTO preferentially binds to intronic regions of pre-mRNA and the depletion of FTO in HEK293T and mouse 3T3-L1 cells also results in changes in pre-mRNA splicing (Zhao et al., 2014; Bartosovic et al., 2017). For m^6^A readers, nuclear reader YTHDC1 regulates splicing of m^6^A-methylated mRNAs by recruiting splicing factors (SRSF3 and SRSF10) (Xiao et al., 2016); HNRNPA2B1, another nuclear reader of m^6^A, elicits consequences on alternative splicing similar to those of METTL3 (Alarcon et al., 2015); HNRNPC and HNRNPG regulate the expression as well as alternative splicing of the target mRNAs via m^6^A-switch (Liu et al., 2015, 2017). A more recent study has reported that m^6^A is decorated in nascent RNA and can regulate the kinetics of RNA splicing (Louloupi et al., 2018). Despite the above rich information supporting a role of m^6^A in splicing, there is one study claiming that mRNA m^6^A modification can be deposited before splicing but it is not required for splicing in mESCs (Ke et al., 2017). Thus, future investigations are still needed to determine how m^6^A directly or indirectly affects pre-mRNA splicing and which transcripts are regulated by m^6^A in different biological contexts.

m^6^A has intricate functions during diverse viral infection. m^6^A can be deposited in the RNAs of Zika virus (ZIKV), hepatitis C virus (HCV), influenza A virus (IAV), simian virus 40 (SV40), and human immunodeficiency virus 1 (HIV-1). m^6^A negatively regulates the infection of ZIKV and HCV (Gokhale et al., 2016; Lichinchi et al., 2016b); while m^6^A promotes gene expression and replication of IAV and SV40 (Courtney et al., 2017; Tsai et al., 2018). The contention for the regulatory roles of m^6^A was observed in HIV: m^6^A was shown to enhance HIV-1 gene expression and replication (Kennedy et al., 2016; Lichinchi et al., 2016a), while m^6^A was also found to inhibit HIV-1 infection by decreasing the reverse transcription (RT) of HIV-1 (Tirumuru et al., 2016; Lu et al., 2018). Besides, m^6^A methylation in the mRNA of host cells also has regulatory roles in response to viral infection (Liu et al., 2019d; Wang et al., 2019). Together, m^6^A is an important epitranscriptomic mark for controlling viral infection, but it is still unclear how m^6^A regulates viral infection and why m^6^A has different regulatory outputs towards diverse viruses.

### At the beginning of mRNA: m^6^Am

The first adenosine proximal to 5’ cap is 2’-O-methylated adenosine (Am), which can be further methylated by methyltransferase PCIF1 to form m^6^Am (Akichika et al., 2019; Boulias et al., 2019; Sendinc et al., 2019; Sun et al., 2019) (Fig. 2B). Similar to m^6^A, the N^6^-methyl group of m^6^Am can also be demethylated by FTO (Mauer et al., 2017; Wei et al., 2018) (Fig. 2B). Since m^6^A-specific antibody do not distinguish between m^6^Am and m^6^A, m^6^A/MeRIP-Seq and miCLIP can be used to detect m^6^Am at transcription start sites (TSSs) (Linder et al., 2015; Sun et al., 2019). Recently, a more specific detection method of m^6^Am has also been developed: m^6^Am-Exo-Seq utilizes a 5’ exonuclease to deplete the internal m^6^A-containing RNA fragments and enrich capped 5’ terminus of mRNA, followed immunoprecipitation (IP) with antibody against m^6^A (Sendinc et al., 2019). Nevertheless, all current m^6^Am sequencing technologies still rely on anti-m^6^A antibody, thus further development of unbiased and specific m^6^Am detection methods are still desired to help us to better understand the m^6^Am methylome.

The presence of m^6^Am was originally suggested to alter mRNA stability (Mauer et al., 2017); however, this finding was recently challenged. The transcripts with m^6^Am-cap were purposed with enhanced stabilities in HEK293T cells, and *FTO* knockdown causes a global increase in the expression level of m^6^Am-containing mRNAs (Mauer et al., 2017). However, the authors did not tease out the combinatorial effects of m^6^Am from internal m^6^A. Another study found FTO depletion does not noticeably affect the expression levels of mRNAs containing only m^6^Am in HEK293T cells (Wei et al., 2018). Further studies revealed that the loss of m^6^Am modification in PCIF1 knockout (KO) HEK293T or MEL624 cells does not significantly affect the level of mRNAs with m^6^Am either (Akichika et al., 2019; Sendinc et al., 2019). Controversial observations were also been reported: when mRNAs in the lower and upper half of gene expression were separately examined, only the half-life of m^6^Am-containing mRNAs in the lower half of gene expression were significantly decreased in *PCIF1* KO HEK293T cells (Boulias et al., 2019). On the other hand, it appears that m^6^Am also influences mRNA translation (Akichika et al., 2019; Sendinc et al., 2019). Collectively, the regulatory function of m^6^Am in mRNA is still at its early stage and remains to be fully explored.

### Another well-known methylated adenosine: m^1^A

m^1^A is an isomer of m^6^A, with the methyl group attached to the N^1^ instead of N^6^ position. m^1^A is known to present in tRNA, rRNA, and recently has also been identified in mRNA (Dominissini et al., 2016; Li et al., 2016a, 2017b; Safra et al., 2017). Similar to m^6^A, m^1^A in RNA is a dynamic and reversible modification. The methyltransferase complex TRMT6-TRMT61A is responsible for the installation of a subset of m^1^A in mRNA, while other set of methyltransferases, TRMT61B and TRMT10C catalyze the formation of m^1^A in mitochondrial mRNA (Li et al., 2017b; Safra et al., 2017) (Fig. 2C). The reversal of m^1^A in RNA can be catalyzed by ALKBH1, ALKBH3, and FTO (Dominissini et al., 2016; Li et al., 2016a; Liu et al., 2016; Wei et al., 2018) (Fig. 2C).

Recently, several groups have independently developed transcriptome-wide approaches to map m^1^A methylomes (m^1^A-ID-Seq, m^1^A-Seq, m^1^A-MAP, and m^1^A-Seq-TGIRT) (Dominissini et al., 2016; Li et al., 2016a, 2017b; Safra et al., 2017). During RT, m^1^A causes termination or misincorporation, thus m^1^A sites can be identified at single-base resolution after IP by commercial antibody and sequencing. Moreover, demethylase treatment or Dimroth rearrangement are further used to remove the RT signatures of m^1^A as an additional validation step. m^1^A-MAP identified 473 m^1^A sites in human mRNA (Li et al., 2017b); however, m^1^A-Seq-TGIRT detected only 15 m^1^A sites in human mRNA (Safra et al., 2017), due to its limited sensitivity (Xiong et al., 2018). This is further exemplified by the fact that all the m^1^A sites identified by m^1^A-Seq-TGIRT are included in the more comprehensive m^1^A list by m^1^A-MAP. In addition, independent studies have reported that the m^1^A/A ratio in human mRNA is about 0.01%–0.05% (Dominissini et al., 2016; Li et al., 2016a; Ueda et al., 2017; Xu et al., 2017), supporting the existence of hundreds to thousands of m^1^A sites in mRNA (Dominissini et al., 2016; Li et al., 2016a, 2017b) instead of just a handful sites detected by m^1^A-Seq-TGIRT (Safra et al., 2017). Moreover, in-depth analysis revealed potential reasons that lead to insensitivity of m^1^A-Seq-TGIRT, including severe reads duplication, rRNA contamination, significant RNA degradation, low efficiency of Dimroth reaction, limited sequencing depth, etc. (Xiong et al., 2018). Very recently, new approaches (m^1^A-IP-Seq and m^1^A-quant-Seq) utilizing an evolved reverse transcriptase that reads through m^1^A more efficiently also reported hundreds of m^1^A sites, further corroborating its prevalence in mRNA (Zhou et al., 2019).

### Chemical modifications in cytosine: m^5^C, hm^5^C, and ac^4^C

m^5^C is formed by methylation at the C^5^ position of cytosine, which is present in tRNA, rRNA, and mRNA (Dubin and Taylor, 1975). In mRNA, NSUN2 is the main m^5^C methyltransferase (Squires et al., 2012; Hussain et al., 2013b; Yang et al., 2017) (Fig. 2D). Drawing lessons from m^5^dC detection in DNA, m^5^C in RNA can be detected by a modified bisulfite treatment to achieve single-base resolution (Schaefer et al., 2009; Squires et al., 2012). To avoid potentially annealing to the inefficiently deaminated RNA templates, ACT random hexamers devoid of Gs were applied to prime the bisulfite-treated poly(A)-enriched RNA samples for RT (Yang et al., 2017). The mRNA export adapter ALYREF and the DNA/RNA binding protein YBX1 have been identified as m^5^C readers (Yang et al., 2017, 2019; Chen et al., 2019). Besides, several groups independently developed strategies to detect m^5^C: Aza-IP utilized a cytidine analogue, 5-azacytidine, to form a covalent adduct with methyltransferase, which can enrich and subsequently sequence m^5^C targets (Khoddami and Cairns, 2013); miCLIP of m^5^C (different from m^6^A miCLIP) exploited the formation of covalent bond between C271A mutant NSUN2 and substrate to detect the enriched m^5^C targets (Hussain et al., 2013b); m^5^C-RIP used m^5^C-specific antibody to identify m^5^C peaks in bacteria, archaea, yeast and plant transcriptomes (Edelheit et al., 2013; Cui et al., 2017b). Among them, bisulfite sequencing is the most widely used, which is single-base resolution and potentially quantitative. However, it also has limitations: it could lead to the loss of RNA due to harsh chemical and thermal conditions, thus this method is insensitive to detect m^5^C in low abundant RNA. Unconverted cytosines and other cytosine modifications resistant to bisulfite treatment may result in false-positive detection (Hussain et al., 2013a; Gilbert et al., 2016; Shafik et al., 2016). Furthermore, Aza-IP and miCLIP of m^5^C are bisulfite-independent and can pre-enrich m^5^C targets, but require over-expression of methyltransferase, which may lead to false-positive detection from nonspecific targeting by the highly expressed and potential mis-localized enzymes within the cell. Therefore, future development of more sensitive and accurate m^5^C detection methods are still desired (Yuan et al., 2019).

m^5^C can be further oxidized by ten-eleven translocation (TET) family enzymes to form hm^5^C (Fu et al., 2014; Delatte et al., 2016; Shen et al., 2018) (Fig. 2E). Similar to m^5^C-RIP, hMeRIP-Seq relied on anti-hm^5^C antibody to detect over 3,000 hm^5^C peaks in Drosophila mRNA (Delatte et al., 2016). Additionally, the N^4^ position of cytosine can be acetylated by the acetyltransferase NAT10 to form ac^4^C, which is present in tRNA, rRNA, and mRNA (Dong et al., 2016; Arango et al., 2018) (Fig. 2F). Based on ac^4^C-specific antibody, acRIP-Seq exploited anti-ac^4^C antibody and identified over 4,000 ac^4^C peaks in the human transcriptome (Arango et al., 2018). However, both detection strategies of hm^5^C and ac^4^C are based on specific antibodies and cannot reach single-base resolution, which hinders functional studies of RNA modification. Thus, learning from the success of single-base and quantitative m^6^A sequencing technologies, optimized methods are expected to be developed (Yuan et al., 2019).

### The rotation isomerization of uridine: Ψ

Ψ, known as the “fifth nucleotide” of RNA, is the most abundant modification in RNA and widely present in tRNA, rRNA, snRNA, and mRNA (Karijolich et al., 2015). The formation of Ψ is catalyzed by two kinds of pseudouridine synthases (PUSs): “stand-alone” PUSs that require no cofactor and the RNA-dependent PUSs that require the cofactor, box H/ACA-box small nucleolar RNA (snoRNA), as guides to recognize substrates (Song and Yi, 2019) (Fig. 2G). In human, stand-alone synthases PUS1, PUS7, TRUB1 and the RNA-dependent synthase DKC1 have been reported to catalyze a subset of Ψ in mRNA (Carlile et al., 2014; Schwartz et al., 2014a; Li et al., 2015), but it is still unclear whether other PUSs can also modify mRNA.

High-throughput sequencing methods for Ψ (Ψ-Seq, Pseudo-Seq, PSI-Seq, and CeU-Seq) rely on a chemical, N-cyclohexyl-N’-β-(4-methylmorpholinium) ethylcarbodiimide (CMC), which can specifically label Ψ (Carlile et al., 2014; Lovejoy et al., 2014; Schwartz et al., 2014a; Li et al., 2015). During RT, the CMC-Ψ adduct can cause stop at one nucleotide 3′ to the labeled Ψ site, enabling the detection of 100–400 Ψ sites in human mRNA at base resolution (Carlile et al., 2014; Lovejoy et al., 2014; Schwartz et al., 2014a). However, these methods cannot pre-enrich Ψ sites and may dropout Ψ in low abundant RNA. CeU-Seq utilized a CMC derivative, azido-CMC (N_3_-CMC), to allow the pre-enrichment of Ψ-containing RNA through biotin pulldown, which identified about 2,000 Ψ sites in human mRNA (Li et al., 2015). In fact, the ratio of Ψ/U in mammalian mRNA as measured by LC-MS/MS (about 0.2%–0.6%) is comparable to the content of m^6^A (Li et al., 2015), which further supports the existence of thousands of Ψ sites in mRNA. The CMC chemistry can also be coupled to high resolution qPCR analysis to conveniently detect locus-specific Ψ sites in mRNA and lncRNA (Lei and Yi, 2017). Moreover, bisulfite treatment can have Ψ nucleotide to form a monobisulfite adduct, which causes a deletion signature at the Ψ sites during RT. Thus, utilizing bisulfite treatment, RBS-Seq has been developed to detect Ψ modification (Khoddami et al., 2019). However, similar to the ordinary CMC labeling, this method also cannot pre-enrich Ψ sites and identified 322 Ψ sites in mRNA; even for abundant tRNA, RBS-Seq failed to detect all known Ψ sites. Recently, by combining CMC-labeling and demethylase treatment, DM-Ψ-Seq has been developed to detect global Ψ sites in tRNAome (Song et al., 2019).

CMC-labeling is not perfect. Alkaline treatment step could lead to RNA degradation, and not all Ψ sites can be equally labeled. These may have led to the low overlap of identified Ψ sites in mRNA by different methods. Yet, Ψ sites in abundant non-coding RNAs (rRNA, etc.) were highly correlated, suggesting the abundance and thus the sequencing depth certainly influence the modification list. Further comparisons of different methods have revealed other factors that need to be considered, such as varied sequencing depth, different bioinformatics algorithms and cutoffs, distinct cell lines and/or growth conditions, etc (Li et al., 2016b; Zaringhalam and Papavasiliou, 2016). On the other hand, considering the dynamic nature of Ψ modification, it is likely that only a subset of pseudouridylation events have been reported. Thus, further improvements for Ψ profiling with quantification and higher sensitivity are still needed.

### Not only a cap modification, but also an internal modification: m^7^G

m^7^G is a well-known mRNA cap modification. It is also prevalent in tRNA and recently has been identified in mRNA as well (Chu et al., 2018; Malbec et al., 2019; Zhang et al., 2019a). METTL1-WDR4, known as a tRNA m^7^G methyltransferase complex, installs a subset of internal m^7^G in mRNA (Fig. 2H). Both antibody-based and chemical labeling sequencing methods have been developed to map m^7^G methylomes. m^7^G-MeRIP-Seq used m^7^G-specific antibody to identified over 2,000 internal m^7^G peaks in the mammalian transcriptome (Zhang et al., 2019a). m^7^G miCLIP-Seq utilized cross-linking-induced truncation and mutation to detect m^7^G (Malbec et al., 2019). m^7^G-Seq adopted a reduction-induced depurination reaction to generate a basic site at m^7^G positions, which can be further labeled with biotin and subsequently pulled down. The labeled m^7^G sites in RNA can cause misincorporation during RT, thus achieving the base-resolution map of m^7^G methylome (Zhang et al., 2019a). Benefiting from high-throughput detection strategies, two groups independently found that internal m^7^G in mRNA plays regulatory roles in translation (Malbec et al., 2019; Zhang et al., 2019a). Considering that METTL1-modified m^7^G in tRNA is also required for translation and modification enzymes are shared between mRNA and tRNA (Lin et al., 2018), it would be interesting to separately probe its function in mRNA.

## Long-read sequencing for DNA and RNA modifications

Most sequencing methods described above work with next-generations equencing, which is limited by short sequencing length. In contrast, third-generation sequencing methods including PacBio Single-Molecule Real-Time (SMRT) sequencing (Ardui et al., 2018; Wenger et al., 2019) and Oxford Nanopore sequencing (Clarke et al., 2009; Jain et al., 2018), have been developed to enable long-read and single-molecule sequencing of DNA and RNA. Apart from the much longer read-length, both SMRT and Nanopore sequencing also allow direct readout of DNA and RNA modification.

SMRT sequencing, which is based on the differentiation of nucleobases in DNA through the fluorescent labelled nucleotide being incorporated into DNA by polymerases, can also detect base modifications using on polymerase kinetics, such as 5mC, 5hmC and 6mA (Flusberg et al., 2010). Genome-wide mapping of 5hmC at single-base resolution in mESCs was realized by chemical labeling-mediated SMRT sequencing (Song et al., 2011a). Chemical labeling enables the affinity enrichment of 5hmC-containing DNA fragments and increases the kinetic signal of 5hmC during SMRT sequencing. SMRT sequencing can detect 6mA in DNA, however, causions should be made since it overestimates 6mA level in DNA samples when it is rare (O’Brown et al., 2019). Moreover, it is possible to detect m^6^A in RNA and secondary structure of RNA by SMRT sequencing combined with reverse transcription (Vilfan et al., 2013).

As for Oxford Nanopore sequencing, different molecules can generate different ionic current when they pass through the nanoscale pore, which is then employed as characterized signatures to discriminate nucleosides in DNA or RNA (Venkatesan and Bashir, 2011; Jain et al., 2016; Garalde et al., 2018). Nanopore sequencing can directly detect DNA or RNA without PCR amplification or cDNA conversion in real time (Rand et al., 2017; Simpson et al., 2017; Garalde et al., 2018). It can be applied to detect different kinds of modified bases, such as 5mC, 5hmC and 6mA in DNA and m^6^A, Inosine, m^5^C, Ψ, and m^7^G in RNA, as well as secondary structure of RNA and G-quadruplex (Li et al., 2013; Simpson et al., 2017; Garalde et al., 2018; Wongsurawat et al., 2018; Liu et al., 2019a; Smith et al., 2019; Viehweger et al., 2019; Workman et al., 2019).

Although third generation sequencing is promising in direct detecting DNA and RNA modification, the high error rate and unmatured base-calling prevent the practical application at present. The combination of certain sequencing methods mentioned above with third generation sequencing could provide highly accurate long-read epigenetic sequencing, such as lrTAPS (Liu et al., 2020).

## Conclusion and outlook

In summary, we highlight the advances of mapping methods for DNA and RNA modification, and biological discoveries with their application in recent years. Collectively, these methods set a stage for systematic investigation of the functional significance of DNA and RNA modification in biological processes and human diseases. However, the current pace of advancement needs to continue in order to develop affordable and accurate assays to detect DNA and RNA modification, especially at the most phenotypically relevant sites, with the eventual goal of bringing these assays to routine use in clinical utility.

## Abbreviations

5caC, 5-carboxylcytosine; 5fC, 5-formylcytosine; 5gmC, 5-(β-glucosyloxymethyl) cytosine; 5hmC, 5-hydroxymethylcytosine; 5mC, 5-methylcytosine; 6mA, N^6^-methyladenine; ac^4^C, N^4^-acetylcytidine; ACE-Seq, APOBEC-coupled epigenetic sequencing; AI, azido derivative of 1,3-indandione; AID/APOBEC, activation-induced (cytidine) deaminase/apolipoprotein B mRNA editing enzyme; AMD-Seq, APOBEC3A-mediated deamination sequencing; APOBEC3A, apolipoprotein B mRNA editing enzyme, catalytic polypeptide-like; BER, base excision repair; BS, bisulfite sequencing; C, cytosine; CAB-Seq, chemical modification-assisted bisulfite sequencing; CLEVER-Seq, chemical-labeling-enabled C-to-T conversion sequencing; CMC, N-cyclohexyl-N’-β-(4-methylmorpholinium) ethylcarbodiimide; DHU, dihydrouracil; DIP-CAB-Seq, DNA immunoprecipitation-coupled CAB-Seq; DNATS, DNA methyltransferases; EM-Seq, Enzymatic Methyl-Seq; EDC, 1-ethyl-3-[3-imethylaminopropyl] carbodiimide hydrochloride; EtONH2, *O*-ethylhydroxylamine; fCAB-Seq, 5fC chemically assisted bisulfite sequencing; fC-CET, 5fC cyclization-enabled C-to-T transition; FFPE, formalin-fixed paraffin embedded; hm^5^C, 5-hydroxymethylcytosine; hmC-CATCH, chemical-assistant C-to-T conversion of 5hmC sequencing; IP, immunoprecipitation; K_2_RuO_4_, potassium ruthenate; KRuO4, potassium perruthenate; lncRNA, long noncoding RNA; lrTAPs, long-read TAPS; m^1^A, N^1^-methyladenosine; m^6^A, N^6^-methyladenosine; m^6^Am, N^6^,2’-O-dimethyladenosine; m^5^C, 5-methylcytosine; m^7^G, N^7^-methylguanosine; MAB-Seq, M.SssI methylase-assisted bisulfite sequencing; MeDIP, Methylated DNA immunoprecipitation; mESCs, mouse embryonic stem cells, mRNA, messenger RNA; NaBH4, sodium borohydride; oxBS-Seq, Oxidative bisulfite sequencing; PCR, polymerase chain reaction; rRNA, ribosomal RNA; RT, reverse transcription; SMRT, Pac Bio Single-Molecule Real-Time; snRNA, small nuclear RNA; snoRNA, small nucleolar RNA; T, thymine; TAB-Seq, TET-assisted bisulfite sequencing; TAPS, TET-assisted pyridine borane sequencing; TDG, thymine DNA glycosylase; Tdg KO mESCs, Tdg Knowkout mESCs; TET1, ten-eleven translocation 1; tRNA, transfer RNA; U, Uracil; WGBS, Whole-genome bisulfite sequencing; β-GT, β-glucosyltransferase; Ψ, pseudouridine.

## References

[CR1] Adey A, Shendure J (2012). Ultra-low-input, tagmentation-based whole-genome bisulfite sequencing. Genome Res.

[CR2] Akichika S, Hirano S, Shichino Y, Suzuki T, Nishimasu H, Ishitani R, Sugita A, Hirose Y, Iwasaki S, Nureki O (2019). Cap-specific terminal N (6)-methylation of RNA by an RNA polymerase II-associated methyltransferase. Science (New York, NY).

[CR3] Alarcon CR, Goodarzi H, Lee H, Liu X, Tavazoie S, Tavazoie SF (2015). HNRNPA2B1 is a mediator of m(6)A-dependent nuclear RNA processing events. Cell.

[CR4] Arango D, Sturgill D, Alhusaini N, Dillman AA, Sweet TJ, Hanson G, Hosogane M, Sinclair WR, Nanan KK, Mandler MD (2018). Acetylation of cytidine in mRNA promotes translation efficiency. Cell.

[CR5] Ardui S, Ameur A, Vermeesch JR, Hestand MS (2018). Single molecule real-time (SMRT) sequencing comes of age: applications and utilities for medical diagnostics. Nucleic Acids Res.

[CR6] Bartosovic M, Molares HC, Gregorova P, Hrossova D, Kudla G, Vanacova S (2017). N6-methyladenosine demethylase FTO targets pre-mRNAs and regulates alternative splicing and 3′-end processing. Nucleic Acids Res.

[CR7] Boccaletto P, Machnicka MA, Purta E, Piatkowski P, Baginski B, Wirecki TK, de Crecy-Lagard V, Ross R, Limbach PA, Kotter A (2018). MODOMICS: a database of RNA modification pathways. 2017 update. Nucleic Acids Res.

[CR8] Bokar JA, Rath-Shambaugh ME, Ludwiczak R, Narayan P, Rottman F (1994). Characterization and partial purification of mRNA N6-adenosine methyltransferase from HeLa cell nuclei. Internal mRNA methylation requires a multisubunit complex. J Biol Chem.

[CR9] Booth MJ, Branco MR, Ficz G, Oxley D, Krueger F, Reik W, Balasubramanian S (2012). Quantitative sequencing of 5-methylcytosine and 5-hydroxymethylcytosine at single-base resolution. Science (New York, NY).

[CR10] Booth MJ, Ost TW, Beraldi D, Bell NM, Branco MR, Reik W, Balasubramanian S (2013). Oxidative bisulfite sequencing of 5-methylcytosine and 5-hydroxymethylcytosine. Nat Protoc.

[CR11] Booth MJ, Marsico G, Bachman M, Beraldi D, Balasubramanian S (2014). Quantitative sequencing of 5-formylcytosine in DNA at single-base resolution. Nat Chem.

[CR12] Booth MJ, Raiber EA, Balasubramanian S (2015). Chemical methods for decoding cytosine modifications in DNA. Chem Rev.

[CR13] Boulias K, Toczydlowska-Socha D, Hawley BR, Liberman N, Takashima K, Zaccara S, Guez T, Vasseur JJ, Debart F, Aravind L (2019). Identification of the m(6)Am methyltransferase PCIF1 reveals the location and functions of m(6)Am in the transcriptome. Mol Cell.

[CR14] Cao G, Li HB (2016). Recent advances in dynamic m6A RNA modification. Open Biol.

[CR15] Carlile TM, Rojas-Duran MF, Zinshteyn B, Shin H, Bartoli KM, Gilbert WV (2014). Pseudouridine profiling reveals regulated mRNA pseudouridylation in yeast and human cells. Nature.

[CR16] Chen K, Lu Z, Wang X, Fu Y, Luo GZ, Liu N, Han D, Dominissini D, Dai Q, Pan T (2015). High-resolution N(6) -methyladenosine (m(6) A) map using photo-crosslinking-assisted m(6) A sequencing. Angew Chem Int Ed Engl.

[CR17] Chen X, Li A, Sun BF, Yang Y, Han YN, Yuan X, Chen RX, Wei WS, Liu Y, Gao CC (2019). 5-methylcytosine promotes pathogenesis of bladder cancer through stabilizing mRNAs. Nat Cell Biol.

[CR18] Chu JM, Ye TT, Ma CJ, Lan MD, Liu T, Yuan BF, Feng YQ (2018). Existence of Internal N7-methylguanosine modification in mRNA determined by differential enzyme treatment coupled with mass spectrometry analysis. ACS Chem Biol.

[CR19] Clark SJ, Smallwood SA, Lee HJ, Krueger F, Reik W, Kelsey G (2017). Genome-wide base-resolution mapping of DNA methylation in single cells using single-cell bisulfite sequencing (scBS-seq). Nat Protoc.

[CR20] Clarke J, Wu HC, Jayasinghe L, Patel A, Reid S, Bayley H (2009). Continuous base identification for single-molecule nanopore DNA sequencing. Nat Nanotechnol.

[CR21] Courtney DG, Kennedy EM, Dumm RE, Bogerd HP, Tsai K, Heaton NS, Cullen BR (2017). Epitranscriptomic enhancement of influenza A virus gene expression and replication. Cell Host Microbe.

[CR22] Cui Q, Shi H, Ye P, Li L, Qu Q, Sun G, Sun G, Lu Z, Huang Y, Yang CG (2017). m(6)A RNA methylation regulates the self-renewal and tumorigenesis of glioblastoma stem cells. Cell Rep.

[CR23] Cui X, Liang Z, Shen L, Zhang Q, Bao S, Geng Y, Zhang B, Leo V, Vardy LA, Lu T (2017). 5-Methylcytosine RNA methylation in *Arabidopsis thaliana*. Molecular plant.

[CR24] Delatte B, Wang F, Ngoc LV, Collignon E, Bonvin E, Deplus R, Calonne E, Hassabi B, Putmans P, Awe S (2016). RNA biochemistry. Transcriptome-wide distribution and function of RNA hydroxymethylcytosine. Science (New York, NY).

[CR25] Dominissini D, Moshitch-Moshkovitz S, Schwartz S, Salmon-Divon M, Ungar L, Osenberg S, Cesarkas K, Jacob-Hirsch J, Amariglio N, Kupiec M (2012). Topology of the human and mouse m6A RNA methylomes revealed by m6A-seq. Nature.

[CR26] Dominissini D, Nachtergaele S, Moshitch-Moshkovitz S, Peer E, Kol N, Ben-Haim MS, Dai Q, Di Segni A, Salmon-Divon M, Clark WC (2016). The dynamic N(1)-methyladenosine methylome in eukaryotic messenger RNA. Nature.

[CR27] Dong C, Niu L, Song W, Xiong X, Zhang X, Zhang Z, Yang Y, Yi F, Zhan J, Zhang H (2016). tRNA modification profiles of the fast-proliferating cancer cells. Biochem Biophys Res Commun.

[CR175] Douvlataniotis K, Bensberg M, Lentini A, Gylemo B, Nestor CE (2020). No evidence for DNA N (6)-methyladenine in mammals. Sci Adv.

[CR28] Dubin DT, Taylor RH (1975). The methylation state of poly A-containing messenger RNA from cultured hamster cells. Nucleic Acids Res.

[CR29] Edelheit S, Schwartz S, Mumbach MR, Wurtzel O, Sorek R (2013). Transcriptome-wide mapping of 5-methylcytidine RNA modifications in bacteria, archaea, and yeast reveals m5C within archaeal mRNAs. PLoS Genet.

[CR30] Ficz G, Branco MR, Seisenberger S, Santos F, Krueger F, Hore TA, Marques CJ, Andrews S, Reik W (2011). Dynamic regulation of 5-hydroxymethylcytosine in mouse ES cells and during differentiation. Nature.

[CR31] Flusberg BA, Webster DR, Lee JH, Travers KJ, Olivares EC, Clark TA, Korlach J, Turner SW (2010). Direct detection of DNA methylation during single-molecule, real-time sequencing. Nat Methods.

[CR32] Frye M, Jaffrey SR, Pan T, Rechavi G, Suzuki T (2016). RNA modifications: what have we learned and where are we headed?. Nat Rev Genet.

[CR33] Fu Y, Jia G, Pang X, Wang RN, Wang X, Li CJ, Smemo S, Dai Q, Bailey KA, Nobrega MA (2013). FTO-mediated formation of N6-hydroxymethyladenosine and N6-formyladenosine in mammalian RNA. Nat Commun.

[CR34] Fu L, Guerrero CR, Zhong N, Amato NJ, Liu Y, Liu S, Cai Q, Ji D, Jin SG, Niedernhofer LJ (2014). Tet-mediated formation of 5-hydroxymethylcytosine in RNA. J Am Chem Soc.

[CR35] Fu Y, Luo GZ, Chen K, Deng X, Yu M, Han D, Hao Z, Liu J, Lu X, Dore LC (2015). N6-methyldeoxyadenosine marks active transcription start sites in Chlamydomonas. Cell.

[CR36] Garalde DR, Snell EA, Jachimowicz D, Sipos B, Lloyd JH, Bruce M, Pantic N, Admassu T, James P, Warland A (2018). Highly parallel direct RNA sequencing on an array of nanopores. Nat Methods.

[CR37] Garcia-Campos MA, Edelheit S, Toth U, Safra M, Shachar R, Viukov S, Winkler R, Nir R, Lasman L, Brandis A (2019). Deciphering the “m(6)A code” via antibody-independent quantitative profiling. Cell.

[CR38] Geula S, Moshitch-Moshkovitz S, Dominissini D, Mansour AA, Kol N, Salmon-Divon M, Hershkovitz V, Peer E, Mor N, Manor YS (2015). Stem cells. m6A mRNA methylation facilitates resolution of naive pluripotency toward differentiation. Science (New York, NY).

[CR39] Gilbert WV, Bell TA, Schaening C (2016). Messenger RNA modifications: form, distribution, and function. Science (New York, NY).

[CR40] Gokhale NS, McIntyre AB, McFadden MJ, Roder AE, Kennedy EM, Gandara JA, Hopcraft SE, Quicke KM, Vazquez C, Willer J (2016). N6-methyladenosine in flaviviridae viral RNA genomes regulates infection. Cell Host Microbe.

[CR41] Greenberg MVC, Bourc’his D (2019). The diverse roles of DNA methylation in mammalian development and disease. Nat Rev Mol Cell Biol.

[CR42] Greer EL, Blanco MA, Gu L, Sendinc E, Liu J, Aristizabal-Corrales D, Hsu CH, Aravind L, He C, Shi Y (2015). DNA methylation on N6-adenine in *C. elegans*. Cell.

[CR43] Guo F, Li X, Liang D, Li T, Zhu P, Guo H, Wu X, Wen L, Gu TP, Hu B (2014). Active and passive demethylation of male and female pronuclear DNA in the mammalian zygote. Cell Stem Cell.

[CR176] Hao Z, Wu T, Cui X, Zhu P, Tan C, Dou X, Hsu KW, Lin YT, Peng PH, Zhang LS (2020). N(6)-deoxyadenosine methylation in mammalian mitochondrial DNA. Mol Cell.

[CR44] Harper JE, Miceli SM, Roberts RJ, Manley JL (1990). Sequence specificity of the human mRNA N6-adenosine methylase in vitro. Nucleic Acids Res.

[CR45] He C (2010). Grand challenge commentary: RNA epigenetics?. Nat Chem Biol.

[CR46] He YF, Li BZ, Li Z, Liu P, Wang Y, Tang Q, Ding J, Jia Y, Chen Z, Li L (2011). Tet-mediated formation of 5-carboxylcytosine and its excision by TDG in mammalian DNA. Science.

[CR47] Hussain S, Aleksic J, Blanco S, Dietmann S, Frye M (2013). Characterizing 5-methylcytosine in the mammalian epitranscriptome. Genome Biol.

[CR48] Hussain S, Sajini AA, Blanco S, Dietmann S, Lombard P, Sugimoto Y, Paramor M, Gleeson JG, Odom DT, Ule J (2013). NSun2-mediated cytosine-5 methylation of vault noncoding RNA determines its processing into regulatory small RNAs. Cell Rep.

[CR49] Ito S, Shen L, Dai Q, Wu SC, Collins LB, Swenberg JA, He C, Zhang Y (2011). Tet proteins can convert 5-methylcytosine to 5-formylcytosine and 5-carboxylcytosine. Science (New York, NY).

[CR50] Jain M, Olsen HE, Paten B, Akeson M (2016). The Oxford Nanopore MinION: delivery of nanopore sequencing to the genomics community. Genome Biol.

[CR51] Jain M, Koren S, Miga KH, Quick J, Rand AC, Sasani TA, Tyson JR, Beggs AD, Dilthey AT, Fiddes IT (2018). Nanopore sequencing and assembly of a human genome with ultra-long reads. Nat Biotechnol.

[CR52] Jia G, Fu Y, Zhao X, Dai Q, Zheng G, Yang Y, Yi C, Lindahl T, Pan T, Yang YG (2011). N6-methyladenosine in nuclear RNA is a major substrate of the obesity-associated FTO. Nat Chem Biol.

[CR53] Karijolich J, Yi C, Yu YT (2015). Transcriptome-wide dynamics of RNA pseudouridylation. Nat Rev Mol Cell Biol.

[CR54] Ke S, Alemu EA, Mertens C, Gantman EC, Fak JJ, Mele A, Haripal B, Zucker-Scharff I, Moore MJ, Park CY (2015). A majority of m6A residues are in the last exons, allowing the potential for 3’ UTR regulation. Genes Dev.

[CR55] Ke S, Pandya-Jones A, Saito Y, Fak JJ, Vagbo CB, Geula S, Hanna JH, Black DL, Darnell JE, Darnell RB (2017). m(6)A mRNA modifications are deposited in nascent pre-mRNA and are not required for splicing but do specify cytoplasmic turnover. Genes Dev.

[CR56] Kennedy EM, Bogerd HP, Kornepati AV, Kang D, Ghoshal D, Marshall JB, Poling BC, Tsai K, Gokhale NS, Horner SM (2016). Posttranscriptional m(6)A editing of HIV-1 mRNAs enhances viral gene expression. Cell Host Microbe.

[CR57] Khoddami V, Cairns BR (2013). Identification of direct targets and modified bases of RNA cytosine methyltransferases. Nat Biotechnol.

[CR58] Khoddami V, Yerra A, Mosbruger TL, Fleming AM, Burrows CJ, Cairns BR (2019). Transcriptome-wide profiling of multiple RNA modifications simultaneously at single-base resolution. Proc Natl Acad Sci USA.

[CR59] Kobayashi H, Kono T (2012). DNA methylation analysis of germ cells by using bisulfite-based sequencing methods. Methods Mol Biol (Clifton, NJ).

[CR60] Kobayashi H, Sakurai T, Miura F, Imai M, Mochiduki K, Yanagisawa E, Sakashita A, Wakai T, Suzuki Y, Ito T (2013). High-resolution DNA methylome analysis of primordial germ cells identifies gender-specific reprogramming in mice. Genome Res.

[CR61] Koch A, Joosten SC, Feng Z, de Ruijter TC, Draht MX, Melotte V, Smits KM, Veeck J, Herman JG, Van Neste L (2018). Author correction: analysis of DNA methylation in cancer: location revisited. Nat Rev Clin Oncol.

[CR62] Kriaucionis S, Heintz N (2009). The nuclear DNA base 5-hydroxymethylcytosine is present in Purkinje neurons and the brain. Science.

[CR63] Lei Z, Yi C (2017). A radiolabeling-free, qPCR-based method for locus-specific pseudouridine detection. Angew Chem Int Ed Engl.

[CR64] Lentini A, Lagerwall C, Vikingsson S, Mjoseng HK, Douvlataniotis K, Vogt H, Green H, Meehan RR, Benson M, Nestor CE (2018). A reassessment of DNA-immunoprecipitation-based genomic profiling. Nat Methods.

[CR65] Li E, Zhang Y (2014). DNA methylation in mammals. Cold Spring Harb Perspect Biol.

[CR66] Li WW, Gong L, Bayley H (2013). Single-molecule detection of 5-hydroxymethylcytosine in DNA through chemical modification and nanopore analysis. Angew Chem Int Ed Engl.

[CR67] Li X, Zhu P, Ma S, Song J, Bai J, Sun F, Yi C (2015). Chemical pulldown reveals dynamic pseudouridylation of the mammalian transcriptome. Nat Chem Biol.

[CR68] Li X, Xiong X, Wang K, Wang L, Shu X, Ma S, Yi C (2016). Transcriptome-wide mapping reveals reversible and dynamic N(1)-methyladenosine methylome. Nat Chem Biol.

[CR69] Li X, Xiong X, Yi C (2016). Epitranscriptome sequencing technologies: decoding RNA modifications. Nat Methods.

[CR70] Li W, Zhang X, Lu X, You L, Song Y, Luo Z, Zhang J, Nie J, Zheng W, Xu D (2017). 5-Hydroxymethylcytosine signatures in circulating cell-free DNA as diagnostic biomarkers for human cancers. Cell Res.

[CR71] Li X, Xiong X, Zhang M, Wang K, Chen Y, Zhou J, Mao Y, Lv J, Yi D, Chen XW (2017). Base-resolution mapping reveals distinct m(1)A methylome in nuclear- and mitochondrial-encoded transcripts. Mol Cell.

[CR72] Li Z, Weng H, Su R, Weng X, Zuo Z, Li C, Huang H, Nachtergaele S, Dong L, Hu C (2017). FTO plays an oncogenic role in acute myeloid leukemia as a N(6)-methyladenosine RNA demethylase. Cancer Cell.

[CR73] Li QY, Xie NB, Xiong J, Yuan BF, Feng YQ (2018). Single-nucleotide resolution analysis of 5-hydroxymethylcytosine in DNA by enzyme-mediated deamination in combination with sequencing. Anal Chem.

[CR74] Lichinchi G, Gao S, Saletore Y, Gonzalez GM (2016). Dynamics of the human and viral m(6)A RNA methylomes during HIV-1 infection of T cells. Nat Microbiol.

[CR75] Lichinchi G, Zhao BS, Wu Y, Lu Z, Qin Y, He C, Rana TM (2016). Dynamics of human and viral RNA methylation during Zika virus infection. Cell Host Microbe.

[CR76] Lin S, Liu Q, Lelyveld VS, Choe J, Szostak JW, Gregory RI (2018). Mettl1/Wdr4-mediated m(7)G tRNA methylome is required for normal mRNA translation and embryonic stem cell self-renewal and differentiation. Mol Cell.

[CR77] Linder B, Grozhik AV, Olarerin-George AO, Meydan C, Mason CE, Jaffrey SR (2015). Single-nucleotide-resolution mapping of m6A and m6Am throughout the transcriptome. Nat Methods.

[CR78] Liu J, Yue Y, Han D, Wang X, Fu Y, Zhang L, Jia G, Yu M, Lu Z, Deng X (2014). A METTL3-METTL14 complex mediates mammalian nuclear RNA N6-adenosine methylation. Nat Chem Biol.

[CR79] Liu N, Dai Q, Zheng G, He C, Parisien M, Pan T (2015). N(6)-methyladenosine-dependent RNA structural switches regulate RNA-protein interactions. Nature.

[CR80] Liu F, Clark W, Luo G, Wang X, Fu Y, Wei J, Wang X, Hao Z, Dai Q, Zheng G (2016). ALKBH1-mediated tRNA demethylation regulates translation. Cell.

[CR81] Liu N, Zhou KI, Parisien M, Dai Q, Diatchenko L, Pan T (2017). N6-methyladenosine alters RNA structure to regulate binding of a low-complexity protein. Nucleic Acids Res.

[CR82] Liu H, Begik O, Lucas MC, Ramirez JM, Mason CE, Wiener D, Schwartz S, Mattick JS, Smith MA, Novoa EM (2019). Accurate detection of m6A RNA modifications in native RNA sequences. Nat Commun.

[CR83] Liu J, Harada BT, He C (2019). Regulation of gene expression by N(6)-methyladenosine in cancer. Trends Cell Biol.

[CR84] Liu J, Li K, Cai J, Zhang M, Zhang X, Xiong X, Meng H, Xu X, Huang Z, Peng J (2019). Landscape and regulation of m(6)A and m(6)Am methylome across human and mouse tissues. Mol Cell.

[CR85] Liu Y, You Y, Lu Z, Yang J, Li P, Liu L, Xu H, Niu Y, Cao X (2019). N (6)-methyladenosine RNA modification-mediated cellular metabolism rewiring inhibits viral replication. Science (New York, NY).

[CR86] Liu Y, Cheng J, Siejka-Zielinska P, Weldon C, Roberts H, Lopopolo M, Magri A, D’Arienzo V, Harris JM, McKeating JA (2020). Accurate targeted long-read DNA methylation and hydroxymethylation sequencing with TAPS. Genome Biol.

[CR87] Louloupi A, Ntini E, Conrad T, Orom UAV (2018). Transient N-6-methyladenosine transcriptome sequencing reveals a regulatory role of m6A in splicing efficiency. Cell Rep.

[CR88] Lovejoy AF, Riordan DP, Brown PO (2014). Transcriptome-wide mapping of pseudouridines: pseudouridine synthases modify specific mRNAs in *S. cerevisiae*. PLoS ONE.

[CR89] Lu X, Song CX, Szulwach K, Wang Z, Weidenbacher P, Jin P, He C (2013). Chemical modification-assisted bisulfite sequencing (CAB-Seq) for 5-carboxylcytosine detection in DNA. J Am Chem Soc.

[CR90] Lu X, Han D, Zhao BS, Song CX, Zhang LS, Dore LC, He C (2015). Base-resolution maps of 5-formylcytosine and 5-carboxylcytosine reveal genome-wide DNA demethylation dynamics. Cell Res.

[CR91] Lu W, Tirumuru N, St Gelais C, Koneru PC, Liu C, Kvaratskhelia M, He C, Wu L (2018). N(6)-methyladenosine-binding proteins suppress HIV-1 infectivity and viral production. J Biol Chem.

[CR92] Malbec L, Zhang T, Chen YS, Zhang Y, Sun BF, Shi BY, Zhao YL, Yang Y, Yang YG (2019). Dynamic methylome of internal mRNA N(7)-methylguanosine and its regulatory role in translation. Cell Res.

[CR93] Mauer J, Luo X, Blanjoie A, Jiao X, Grozhik AV, Patil DP, Linder B, Pickering BF, Vasseur JJ, Chen Q (2017). Reversible methylation of m(6)Am in the 5′ cap controls mRNA stability. Nature.

[CR94] Meyer KD (2019). DART-seq: an antibody-free method for global m(6)A detection. Nat Methods.

[CR95] Meyer KD, Saletore Y, Zumbo P, Elemento O, Mason CE, Jaffrey SR (2012). Comprehensive analysis of mRNA methylation reveals enrichment in 3’ UTRs and near stop codons. Cell.

[CR96] Molinie B, Wang J, Lim KS, Hillebrand R, Lu ZX (2016). m(6)A-LAIC-seq reveals the census and complexity of the m(6)A epitranscriptome. Nat Methods.

[CR177] Musheev MU, Baumgärtner A, Krebs L, Niehrs C (2020). The origin of genomic N^6^-methyl-deoxyadenosine in mammalian cells. Nat Chem Biol.

[CR97] Neri F, Incarnato D, Krepelova A, Rapelli S, Anselmi F, Parlato C, Medana C, Dal Bello F, Oliviero S (2015). Single-base resolution analysis of 5-formyl and 5-carboxyl cytosine reveals promoter DNA methylation dynamics. Cell Rep.

[CR98] O’Brown ZK, Boulias K, Wang J, Wang SY, O’Brown NM, Hao Z, Shibuya H, Fady PE, Shi Y, He C (2019). Sources of artifact in measurements of 6mA and 4mC abundance in eukaryotic genomic DNA. BMC Genom.

[CR99] Patil DP, Chen CK, Pickering BF, Chow A, Jackson C, Guttman M, Jaffrey SR (2016). m(6)A RNA methylation promotes XIST-mediated transcriptional repression. Nature.

[CR100] Pendleton KE, Chen B, Liu K, Hunter OV, Xie Y, Tu BP, Conrad NK (2017). The U6 snRNA m(6)A methyltransferase METTL16 regulates SAM synthetase intron retention. Cell.

[CR101] Pfaffeneder T, Hackner B, Truss M, Munzel M, Muller M, Deiml CA, Hagemeier C, Carell T (2011). The discovery of 5-formylcytosine in embryonic stem cell DNA. Angew Chem.

[CR102] Ping XL, Sun BF, Wang L, Xiao W, Yang X, Wang WJ, Adhikari S, Shi Y, Lv Y, Chen YS (2014). Mammalian WTAP is a regulatory subunit of the RNA N6-methyladenosine methyltransferase. Cell Res.

[CR103] Raiber E-A, Hardisty R, van Delft P, Balasubramanian S (2017). Mapping and elucidating the function of modified bases in DNA. Nat Rev Chem.

[CR104] Rand AC, Jain M, Eizenga JM, Musselman-Brown A, Olsen HE, Akeson M, Paten B (2017). Mapping DNA methylation with high-throughput nanopore sequencing. Nat Methods.

[CR105] Roundtree IA, Evans ME, Pan T, He C (2017). Dynamic RNA modifications in gene expression regulation. Cell.

[CR106] Safra M, Sas-Chen A, Nir R, Winkler R, Nachshon A, Bar-Yaacov D, Erlacher M, Rossmanith W, Stern-Ginossar N, Schwartz S (2017). The m1A landscape on cytosolic and mitochondrial mRNA at single-base resolution. Nature.

[CR107] Schaefer M, Pollex T, Hanna K, Lyko F (2009). RNA cytosine methylation analysis by bisulfite sequencing. Nucleic Acids Res.

[CR108] Schutsky EK, DeNizio JE, Hu P, Liu MY, Nabel CS, Fabyanic EB, Hwang Y, Bushman FD, Wu H, Kohli RM (2018). Nondestructive, base-resolution sequencing of 5-hydroxymethylcytosine using a DNA deaminase. Nat Biotechnol.

[CR109] Schwartz S, Agarwala SD, Mumbach MR, Jovanovic M, Mertins P, Shishkin A, Tabach Y, Mikkelsen TS, Satija R, Ruvkun G (2013). High-resolution mapping reveals a conserved, widespread, dynamic mRNA methylation program in yeast meiosis. Cell.

[CR110] Schwartz S, Bernstein DA, Mumbach MR, Jovanovic M, Herbst RH, Leon-Ricardo BX, Engreitz JM, Guttman M, Satija R, Lander ES (2014). Transcriptome-wide mapping reveals widespread dynamic-regulated pseudouridylation of ncRNA and mRNA. Cell.

[CR111] Schwartz S, Mumbach Maxwell R, Jovanovic M, Wang T, Maciag K, Bushkin GG, Mertins P, Ter-Ovanesyan D, Habib N, Cacchiarelli D (2014). Perturbation of m6A writers reveals two distinct classes of mRNA methylation at internal and 5′ sites. Cell Rep.

[CR112] Sendinc E, Valle-Garcia D, Dhall A, Chen H, Henriques T, Navarrete-Perea J, Sheng W, Gygi SP, Adelman K, Shi Y (2019). PCIF1 catalyzes m6Am mRNA methylation to regulate gene expression. Mol Cell.

[CR113] Shafik A, Schumann U, Evers M, Sibbritt T, Preiss T (2016). The emerging epitranscriptomics of long noncoding RNAs. Biochim Biophys Acta.

[CR114] Shen L, Wu H, Diep D, Yamaguchi S, D’Alessio AC, Fung HL, Zhang K, Zhang Y (2013). Genome-wide analysis reveals TET- and TDG-dependent 5-methylcytosine oxidation dynamics. Cell.

[CR115] Shen Q, Zhang Q, Shi Y, Shi Q, Jiang Y, Gu Y, Li Z, Li X, Zhao K, Wang C (2018). Tet2 promotes pathogen infection-induced myelopoiesis through mRNA oxidation. Nature.

[CR116] Shirane K, Toh H, Kobayashi H, Miura F, Chiba H, Ito T, Kono T, Sasaki H (2013). Mouse oocyte methylomes at base resolution reveal genome-wide accumulation of non-CpG methylation and role of DNA methyltransferases. PLoS Genet.

[CR178] Shu X, Cao J, Cheng M, Xiang S, Gao M, Li T, Ying X, Wang F, Yue Y, Lu Z (2020). A metabolic labeling method detects m^6^A transcriptome-wide at single base resolution. Nat Chem Biol.

[CR117] Simpson JT, Workman RE, Zuzarte PC, David M, Dursi LJ, Timp W (2017). Detecting DNA cytosine methylation using nanopore sequencing. Nat Methods.

[CR118] Smallwood SA, Lee HJ, Angermueller C, Krueger F, Saadeh H, Peat J, Andrews SR, Stegle O, Reik W, Kelsey G (2014). Single-cell genome-wide bisulfite sequencing for assessing epigenetic heterogeneity. Nat Methods.

[CR119] Smith AM, Jain M, Mulroney L, Garalde DR, Akeson M (2019). Reading canonical and modified nucleobases in 16S ribosomal RNA using nanopore native RNA sequencing. PLoS ONE.

[CR120] Song J, Yi C (2019). Reading chemical modifications in the transcriptome. J Mol Biol.

[CR121] Song CX, Clark TA, Lu XY, Kislyuk A, Dai Q, Turner SW, He C, Korlach J (2011). Sensitive and specific single-molecule sequencing of 5-hydroxymethylcytosine. Nat Methods.

[CR122] Song CX, Szulwach KE, Fu Y, Dai Q, Yi C, Li X, Li Y, Chen CH, Zhang W, Jian X (2011). Selective chemical labeling reveals the genome-wide distribution of 5-hydroxymethylcytosine. Nat Biotechnol.

[CR123] Song CX, Szulwach KE, Dai Q, Fu Y, Mao SQ, Lin L, Street C, Li Y, Poidevin M, Wu H (2013). Genome-wide profiling of 5-formylcytosine reveals its roles in epigenetic priming. Cell.

[CR124] Song CX, Yin S, Ma L, Wheeler A, Chen Y, Zhang Y, Liu B, Xiong J, Zhang W, Hu J (2017). 5-Hydroxymethylcytosine signatures in cell-free DNA provide information about tumor types and stages. Cell Res.

[CR125] Song J, Zhuang Y, Zhu C, Meng H, Lu B, Xie B, Peng J, Li M, Yi C (2019). Differential roles of human PUS10 in miRNA processing and tRNA pseudouridylation. Nat Chem Biol.

[CR126] Squires JE, Patel HR, Nousch M, Sibbritt T, Humphreys DT, Parker BJ, Suter CM, Preiss T (2012). Widespread occurrence of 5-methylcytosine in human coding and non-coding RNA. Nucleic Acids Res.

[CR127] Stroud H, Feng S, Morey Kinney S, Pradhan S, Jacobsen SE (2011). 5-Hydroxymethylcytosine is associated with enhancers and gene bodies in human embryonic stem cells. Genome Biol.

[CR128] Su R, Dong L, Li C, Nachtergaele S, Wunderlich M, Qing Y, Deng X, Wang Y, Weng X, Hu C (2018). R-2HG exhibits anti-tumor activity by targeting FTO/m(6)A/MYC/CEBPA signaling. Cell.

[CR129] Sun H, Zhang M, Li K, Bai D, Yi C (2019). Cap-specific, terminal N(6)-methylation by a mammalian m(6)Am methyltransferase. Cell Res.

[CR130] Tahiliani M, Koh KP, Shen Y, Pastor WA, Bandukwala H, Brudno Y, Agarwal S, Iyer LM, Liu DR, Aravind L (2009). Conversion of 5-methylcytosine to 5-hydroxymethylcytosine in mammalian DNA by MLL partner TET1. Science.

[CR131] Tanaka K, Okamoto A (2007). Degradation of DNA by bisulfite treatment. Bioorg Med Chem Lett.

[CR132] Tirumuru N, Zhao BS, Lu W, Lu Z, He C, Wu L (2016). N(6)-methyladenosine of HIV-1 RNA regulates viral infection and HIV-1 Gag protein expression. Elife.

[CR133] Tsai K, Courtney DG, Cullen BR (2018). Addition of m6A to SV40 late mRNAs enhances viral structural gene expression and replication. PLoS Pathog.

[CR134] Ueda Y, Ooshio I, Fusamae Y, Kitae K, Kawaguchi M, Jingushi K, Hase H, Harada K, Hirata K, Tsujikawa K (2017). AlkB homolog 3-mediated tRNA demethylation promotes protein synthesis in cancer cells. Sci Rep.

[CR135] Unnikrishnan A, Freeman WM, Jackson J, Wren JD, Porter H, Richardson A (2019). The role of DNA methylation in epigenetics of aging. Pharmacol Therapeut.

[CR136] Vaisvila R, Ponnaluri VKC, Sun Z, Langhorst BW, Saleh L, Guan S, Dai N, Campbell MA, Sexton B, Marks K (2019). EM-seq: detection of DNA methylation at single base resolution from picograms of DNA. BioRxiv Dec.

[CR137] Venkatesan BM, Bashir R (2011). Nanopore sensors for nucleic acid analysis. Nat Nanotechnol.

[CR138] Viehweger A, Krautwurst S, Lamkiewicz K, Madhugiri R, Ziebuhr J, Holzer M, Marz M (2019). Direct RNA nanopore sequencing of full-length coronavirus genomes provides novel insights into structural variants and enables modification analysis. Genome Res.

[CR139] Vilfan ID, Tsai YC, Clark TA, Wegener J, Dai Q, Yi C, Pan T, Turner SW, Korlach J (2013). Analysis of RNA base modification and structural rearrangement by single-molecule real-time detection of reverse transcription. J Nanobiotechnol.

[CR140] Wang Y, Li Y, Toth JI, Petroski MD, Zhang Z, Zhao JC (2014). N6-methyladenosine modification destabilizes developmental regulators in embryonic stem cells. Nat Cell Biol.

[CR141] Wang L, Wen M, Cao X (2019). Nuclear hnRNPA2B1 initiates and amplifies the innate immune response to DNA viruses. Science.

[CR179] Wang Y, Xiao Y, Dong S, Yu Q, Jia G (2020). Antibody-free enzyme-assisted chemical approach for detection of N^6^-methyladenosine. Nat Chem Biol.

[CR142] Weber M, Davies JJ, Wittig D, Oakeley EJ, Haase M, Lam WL, Schubeler D (2005). Chromosome-wide and promoter-specific analyses identify sites of differential DNA methylation in normal and transformed human cells. Nat Genet.

[CR143] Wei J, Liu F, Lu Z, Fei Q, Ai Y, He PC, Shi H, Cui X, Su R, Klungland A (2018). Differential m(6)A, m(6)Am, and m(1)A demethylation mediated by FTO in the cell nucleus and cytoplasm. Mol Cell.

[CR144] Wen J, Lv R, Ma H, Shen H, He C, Wang J, Jiao F, Liu H, Yang P, Tan L (2018). Zc3h13 regulates nuclear RNA m(6)A methylation and mouse embryonic stem cell self-renewal. Mol Cell.

[CR145] Wenger AM, Peluso P, Rowell WJ, Chang PC, Hall RJ, Concepcion GT, Ebler J, Fungtammasan A, Kolesnikov A, Olson ND (2019). Accurate circular consensus long-read sequencing improves variant detection and assembly of a human genome. Nat Biotechnol.

[CR146] Wongsurawat T, Jenjaroenpun P, Wassenaar TM, Wadley TD, Wanchai V, Akel NS, Franco AT, Jennings ML, Ussery DW, Nookaew I (2018). Decoding the epitranscriptional landscape from native RNA sequences. bioRxiv.

[CR147] Workman RE, Tang AD, Tang PS, Jain M, Tyson JR, Razaghi R, Zuzarte PC, Gilpatrick T, Payne A, Quick J (2019). Nanopore native RNA sequencing of a human poly(A) transcriptome. Nat Methods.

[CR148] Wu H, Wu X, Shen L, Zhang Y (2014). Single-base resolution analysis of active DNA demethylation using methylase-assisted bisulfite sequencing. Nat Biotechnol.

[CR149] Wu H, Wu X, Zhang Y (2016). Base-resolution profiling of active DNA demethylation using MAB-seq and caMAB-seq. Nat Protoc.

[CR150] Xia B, Han D, Lu X, Sun Z, Zhou A, Yin Q, Zeng H, Liu M, Jiang X, Xie W (2015). Bisulfite-free, base-resolution analysis of 5-formylcytosine at the genome scale. Nat Methods.

[CR151] Xiang Y, Laurent B, Hsu CH, Nachtergaele S, Lu Z, Sheng W, Xu C, Chen H, Ouyang J, Wang S (2017). RNA m(6)A methylation regulates the ultraviolet-induced DNA damage response. Nature.

[CR152] Xiao W, Adhikari S, Dahal U, Chen YS, Hao YJ, Sun BF, Sun HY, Li A, Ping XL, Lai WY (2016). Nuclear m(6)A reader YTHDC1 regulates mRNA splicing. Mol Cell.

[CR153] Xiao S, Cao S, Huang Q, Xia L, Deng M, Yang M, Jia G, Liu X, Shi J, Wang W (2019). The RNA N(6)-methyladenosine modification landscape of human fetal tissues. Nat Cell Biol.

[CR154] Xiong X, Li X, Wang K, Yi C (2018). Perspectives on topology of the human m(1)A methylome at single nucleotide resolution. RNA (New York, NY).

[CR155] Xu L, Liu X, Sheng N, Oo KS, Liang J, Chionh YH, Xu J, Ye F, Gao YG, Dedon PC (2017). Three distinct 3-methylcytidine (m(3)C) methyltransferases modify tRNA and mRNA in mice and humans. J Biol Chem.

[CR156] Yamaguchi S, Hong K, Liu R, Shen L, Inoue A, Diep D, Zhang K, Zhang Y (2012). Tet1 controls meiosis by regulating meiotic gene expression. Nature.

[CR157] Yang X, Yang Y, Sun BF, Chen YS, Xu JW, Lai WY, Li A, Wang X, Bhattarai DP, Xiao W (2017). 5-methylcytosine promotes mRNA export - NSUN2 as the methyltransferase and ALYREF as an m(5)C reader. Cell Res.

[CR158] Yang Y, Wang L, Han X, Yang WL, Zhang M, Ma HL, Sun BF, Li A, Xia J, Chen J (2019). RNA 5-methylcytosine facilitates the maternal-to-zygotic transition by preventing maternal mRNA decay. Mol Cell.

[CR159] Yu M, Hon GC, Szulwach KE, Song CX, Jin P, Ren B, He C (2012). Tet-assisted bisulfite sequencing of 5-hydroxymethylcytosine. Nat Protoc.

[CR160] Yu M, Hon GC, Szulwach KE, Song CX, Zhang L, Kim A, Li X, Dai Q, Shen Y, Park B (2012). Base-resolution analysis of 5-hydroxymethylcytosine in the mammalian genome. Cell.

[CR161] Yu J, Chen M, Huang H, Zhu J, Song H, Zhu J, Park J, Ji SJ (2018). Dynamic m6A modification regulates local translation of mRNA in axons. Nucleic Acids Res.

[CR162] Yuan F, Bi Y, Siejka-Zielinska P, Zhou YL, Zhang XX, Song CX (2019). Bisulfite-free and base-resolution analysis of 5-methylcytidine and 5-hydroxymethylcytidine in RNA with peroxotungstate. Chem Commun (Camb).

[CR163] Yue Y, Liu J, Cui X, Cao J, Luo G, Zhang Z, Cheng T, Gao M, Shu X, Ma H (2018). VIRMA mediates preferential m(6)A mRNA methylation in 3’UTR and near stop codon and associates with alternative polyadenylation. Cell Discov.

[CR164] Zaringhalam M, Papavasiliou FN (2016). Pseudouridylation meets next-generation sequencing. Methods (San Diego, Calif).

[CR165] Zeng H, He B, Xia B, Bai D, Lu X, Cai J, Chen L, Zhou A, Zhu C, Meng H (2018). Bisulfite-free, nanoscale analysis of 5-hydroxymethylcytosine at single base resolution. J Am Chem Soc.

[CR166] Zhang G, Huang H, Liu D, Cheng Y, Liu X, Zhang W, Yin R, Zhang D, Zhang P, Liu J (2015). N6-methyladenine DNA modification in Drosophila. Cell.

[CR167] Zhang LS, Liu C, Ma H, Dai Q, Sun HL, Luo G, Zhang Z, Zhang L, Hu L, Dong X (2019). Transcriptome-wide mapping of internal N(7)-methylguanosine methylome in mammalian mRNA. Mol Cell.

[CR168] Zhang Z, Chen LQ, Zhao YL, Yang CG, Roundtree IA, Zhang Z, Ren J, Xie W, He C, Luo GZ (2019). Single-base mapping of m(6)A by an antibody-independent method. Sci Adv.

[CR169] Zhao X, Yang Y, Sun BF, Shi Y, Yang X, Xiao W, Hao YJ, Ping XL, Chen YS, Wang WJ (2014). FTO-dependent demethylation of N6-methyladenosine regulates mRNA splicing and is required for adipogenesis. Cell Res.

[CR170] Zheng G, Dahl JA, Niu Y, Fedorcsak P, Huang CM, Li CJ, Vagbo CB, Shi Y, Wang WL, Song SH (2013). ALKBH5 is a mammalian RNA demethylase that impacts RNA metabolism and mouse fertility. Mol Cell.

[CR171] Zhou J, Wan J, Gao X, Zhang X, Jaffrey SR, Qian SB (2015). Dynamic m(6)A mRNA methylation directs translational control of heat shock response. Nature.

[CR172] Zhou J, Wan J, Shu XE, Mao Y, Liu XM, Yuan X, Zhang X, Hess ME, Bruning JC, Qian SB (2018). N(6)-methyladenosine guides mRNA alternative translation during integrated stress response. Mol Cell.

[CR173] Zhou H, Rauch S, Dai Q, Cui X, Zhang Z, Nachtergaele S, Sepich C, He C, Dickinson BC (2019). Evolution of a reverse transcriptase to map N(1)-methyladenosine in human messenger RNA. Nat Methods.

[CR174] Zhu C, Gao Y, Guo H, Xia B, Song J, Wu X, Zeng H, Kee K, Tang F, Yi C (2017). Single-Cell 5-formylcytosine landscapes of mammalian early embryos and ESCs at single-base resolution. Cell Stem Cell.

